# QTL analysis for growth and wood properties across multiple pedigrees and sites in Eucalyptus globulus

**DOI:** 10.1186/1753-6561-5-S7-O8

**Published:** 2011-09-13

**Authors:** Jules S Freeman, Brad M Potts, Geoff M Downes, Saravanan Thavamanikumar, David J Pilbeam, Corey J Hudson, Rene E Vaillancourt

**Affiliations:** 1 School of Plant Science and CRC for Forestry, University of Tasmania, Hobart, Tasmania 7001, Australia; 2 CSIRO Sustainable Agriculture Flagship and Cooperative Research Centre for Forestry, Private Bag 12, Hobart, TAS 7001, Australia; 3 Department of Forest and Ecosystem Science and CRC for Forestry, The University of Melbourne, Water Street, Creswick, Victoria 3363, Australia; 4 Southern Tree Breeding Association Inc., 38 Helen Street, PO Box 1811, Mount Gambier, South Australia, 5290, Australia

## Background

*Eucalyptus globulus* is the most widely planted species for pulpwood production in temperate regions of the world and there are breeding programs in numerous countries. There is interest in molecular approaches to breeding, particularly marker assisted selection of wood properties. QTL analysis has an important role in identifying positional candidate genes responsible for variation in wood properties. This is one approach to targeting genes which may harbour functional allelic variants (SNPs). The objective of this study was to detect and validate QTL across multiple sites and pedigrees, in order to identify genomic regions and genes affecting growth and wood properties with wide applicability in the species. We also aimed to determine the proportion of QTL which were stable in their expression across sites of contrasting productivity. Such information will be important to exploit the full potential of the impending *Eucalyptus* genome sequences.

## Methods

Linkage mapping and QTL analysis were conducted in 650 individuals from four separate pedigrees; a clonally replicated outbred F_2_ family grown on a single site (112 genotypes), and three F_1_ families (180 individuals each) grown at two additional widely separated sites. Trees were assessed for growth, wood density, cellulose content, pulp yield, klason lignin content and the syringyl: guaiacyl (S:G) ratio of lignin at 7 years of age. Chemical traits were estimated using NIR spectroscopy [1]. Saturated linkage maps were constructed in each family, predominantly from Diversity Array Technology (DArT) markers [2] as well as some SSRs, candidate genes and AFLP markers using Joinmap. Common markers were used to construct a consensus map of all families which was used for the QTL analyses. QTL analyses were conducted at various levels (across all families and sites combined, in each family and in each family by site combination) in order to investigate the stability of QTL expression across families and sites.

## Results and discussion

Ninety eight QTL were detected across all analyses, of which 87 affected wood properties and 11 affected growth. QTL for several different chemical wood properties were co-located, consistent with their high phenotypic correlations. Several of the QTL detected co-located with previously reported QTL in *E. nitens*; *E. globulus*; and *E.grandis*[3-5] and candidate genes including COBL4 [5,6], and CCR [4]. Major QTL were also identified in regions of the genome where no candidate genes or QTL had been previously mapped. Nineteen of the QTL were significant in more than one family, and therefore validated. These validated QTL would be the priority for searches for positional candidate genes in the genome sequence of *Eucalyptus grandis*.

Significant QTL by environment interaction was found for 17 out of the 98 (17%) wood property QTL and for 5 out of 11 (45%) growth QTL. This was consistent with significant family by site interactions exhibited across these sites, particularly for growth. The higher level of QTL by environment interaction for growth compared to wood properties was consistent with quantitative genetic studies in the genus [7]. Nevertheless, there was a surprising proportion of wood property QTL (including validated QTL) which were expressed on one site, but not the other within the same family. While there are few multi-site QTL or association mapping studies in forest trees, in most such studies high levels of QTL (or SNP) by environment interactions have been found [8,9].

## Conclusions

Comparative mapping demonstrates that the genomes of commercially important eucalypt species (within Symphyomyrtus) are highly syntenic and co-linear (e.g. Hudson in prep), suggesting QTL results may be applicable across eucalypt species. It is therefore important to use transferable markers such as DArTs and SSRs to facilitate comparison of QTL results between studies. Considering the findings of this and previous QTL studies in the genus, growth and wood properties appear to be highly polygenic. Several of the QTL detected co-located with previously reported QTL in *E. nitens*; *E. globulus*; and *E.grandis* and also with some well characterised wood property candidate genes. However, the specificity of many QTL to particular families and sites in this study reinforces the importance of considering the effect of site and genetic setting may complicate the application of marker assisted selection (MAS) or genomic selection.
